# Drowning is an apparent and unexpected recurrent cause of mass mortality of Common starlings (*Sturnus vulgaris*)

**DOI:** 10.1038/srep17020

**Published:** 2015-11-25

**Authors:** Becki Lawson, J. Paul Duff, Katie M. Beckmann, Julian Chantrey, Kirsi M. Peck, Richard M. Irvine, Robert A. Robinson, Andrew A. Cunningham

**Affiliations:** 1 Institute of Zoology, Zoological Society of London, Regents Park, London, NW1 4RY, UK; 2 Animal & Plant Health Agency, Penrith, Cumbria, CA11 9RR, UK; 3 University of Liverpool, Leahurst Campus Neston, South Wirral CH64 7TE, UK; 4 Royal Society for the Protection of Birds, The Lodge, Sandy, Bedfordshire, SG19 2DL, UK; 5 Animal & Plant Health Agency (APHA), APHA Weybridge, New Haw, Addlestone, Surrey, KT15 3NB, UK; 6 British Trust for Ornithology, The Nunnery, Thetford, Norfolk, IP24 2PU, UK

## Abstract

Drowning is infrequently reported as a cause of death of wild birds and such incidents typically involve individual, rather than multiple, birds. Over a 21-year period (1993 to 2013 inclusive), we investigated 12 incidents of mortality of multiple (2 − 80+) Common starlings (*Sturnus vulgaris*) in Great Britain that appeared to be due to drowning. More than ten birds were affected in ten of these reported incidents. These incidents always occurred during the spring and early summer months and usually involved juvenile birds. In all cases, circumstantial evidence and post-mortem examinations indicated drowning to be the most likely cause of death with no underlying disease found. A behavioural explanation seems likely, possibly related to the gregarious nature of this species combined with juvenile inexperience in identifying water hazards. A review of data from the ringed bird recovery scheme across Great Britain (1909–2013 inclusive) of both starlings and Common blackbirds (*Turdus merula*), also a common garden visitor, identified additional suspected drowning incidents, which were significantly more common in the former species, supporting a species predisposition to drowning. For each species there was a marked seasonal peak from April to August. Drowning should be included as a differential diagnosis when investigating incidents of multiple starling mortality, especially of juveniles.

Mass mortality incidents that involve multiple wild birds attract considerable public concern and media attention. Infectious disease is frequently considered a likely cause of these incidents, and particular focus is placed on the exclusion of notifiable and zoonotic pathogens, such as H5N1 highly pathogenic avian influenza virus^1^ and West Nile virus^2^. Mortality events involving multiple wild birds also can be caused by environmental pollutants, agrochemicals^3^ or avicides and biotoxins, including *Clostridium botulinum* toxins^4^, ethanol^5^,^6^ and cyanobacterial toxins^7^.

Physical causes of mortalities of multiple wild birds include blunt trauma (due to e.g. road traffic accidents, power line collisions, air strikes, predator attacks or disorientation following fireworks or sonic booms)^8^ and adverse weather (hail storms, thunderstorms, lightning)^9^,^10^,^11^.

The Common starling (*Sturnus vulgaris*) is a social flocking bird. In Great Britain (GB), this species has undergone a population decline of over 50% since 1964; the current population is estimated at 3.4 million birds during the breeding season^12^,^13^. There have been few studies of the causes of morbidity and mortality of starlings in GB and these have focused on non-infectious causes including predation, road traffic incidents, shot injury, falling down chimneys and food shortage^14^,^15^. Several infectious diseases have been reported to affect starlings including avian pox^16^,^17^, syngamosis^18^, avian tuberculosis^19^ ^1^ and *Mycoplasma sturni* infection (of unknown clinical significance though detected in association with conjunctivitis in some cases)^20^,^21^. Mortality in multiple birds due to infectious diseases, such as mycoplasmosis^22^, salmonellosis^23^ and trichomonosis^24^, has been frequently diagnosed in some passerine species, particularly finches and sparrows. Salmonellosis is not known to frequently affect starlings in GB, but a large-scale epidemic of salmonellosis occurred in starlings in north-eastern Spain in early 2005^25^. A novel starling circovirus was characterised from the spleen of diseased and healthy starlings in the Spanish population where the epidemic occurred, the clinical significance of which remains uncertain^26^. In GB, diagnosis of infectious disease as a cause of starling mortality is rare and these incidents typically involve individual birds. In contrast, several incidents of mortality involving multiple starlings have been reported in GB due to poisoning^27^ and physical trauma (e.g. air strike^28^, road traffic collisions^29^); the latter is often thought to occur as a result of their flocking behaviour.

Following repeated identification of mass mortalities of starlings associated with water bodies, we reviewed our pathology records to determine cause of death, how commonly apparent drowning of starlings occurred, and to investigate possible risk factors for this. Whilst wild passerines found dead in water bodies are likely to have drowned, pathological examinations are required to exclude alternative causes of death, such as trauma or poisoning, or to determine if the carcass(es) had been immersed in water after death. Drowning refers to death caused by inhalation of water, or other fluids, or asphyxiation following an inability to inhale air due to immersion. Death by drowning is considered a difficult diagnosis to confirm in human forensic medicine^30^. Several signs may be present in birds, including wet or dry matted plumage; frothy fluid in the upper airways or alimentary tract; and water, inhaled diatoms, other plant material, mud or sand in the respiratory tract. The latter observations, whilst not definitive proof, are often considered significant findings highly indicative of drowning^31^,^32^,^33^,^34^. However, confirmation of drowning on post-mortem examination (PME) is often problematic since none of these signs may be present, many of them are transient and only detected in freshly dead animals and some may occur with carcass immersion following death. Consequently, a diagnosis of drowning often remains presumptive and depends on circumstantial evidence and the exclusion of other causes of death^30^,^32^.

Drowning has occasionally been described as a cause of death of terrestrial wild birds in GB, including barn owl (*Tyto alba*), tawny owl (*Strix aluco*), little owl (*Athene noctua*) and kestrel (*Falco tinnunculus*)^35^. A single starling was observed being caught by a female sparrowhawk (*Accipiter nisus*) and taken to a nearby pond where the raptor proceeded to drown its victim^36^. A review of the causes of death reported for starlings ringed in GB and found dead in the 1950s noted that drowning accounted for 1.4% of starling recoveries^15^. There was a seasonal peak during the spring (May-June) in these reports that was mirrored in recoveries due to other traumatic causes of mortality (e.g. fallen down chimney, killed by cat). To the authors’ knowledge, however, drowning has not been reported previously as a cause of starling mortalities involving multiple birds.

To provide a broader perspective, we also reviewed recovery records of birds ringed as part of the British bird-ringing scheme for the period 1909–2013 inclusive^37^. Birds that were found in water bodies and considered likely to have drowned were noted and the frequency of occurrence, the seasonality of these incidents and the types of water bodies involved were quantified. We compared the incidence of such records in starling and Common blackbird (*Turdus merula*) to ascertain whether starlings were particularly susceptible to this cause of mortality, as the blackbird is another passerine of similar body size to the starling that is numerous within garden habitats. The blackbird is commonly encountered bathing and drinking in the same types of water body, so also has the potential to drown in these water bodies, with a similar likelihood of reporting if incidents occur.

The objective of this study was to describe the occurrence of incidents of starling mortality in water bodies and the extent to which these were due to drowning by reviewing available pathology reports and historical bird ring recovery records. Potential explanatory factors leading to these events are discussed.

## Methods

Retrospective reviews were conducted to identify the extent of apparent drowning of starlings and other passerines using two complementary datasets: the first dataset comprised reports of British wild bird mortality incidents and their pathological investigation, 1993–2013, held by the Institute of Zoology (IoZ), Royal Society for the Protection of Birds (RSPB) and Animal Health & Veterinary Laboratories Agency (AHVLA): the second dataset comprised wild bird ring recovery data , 1909–2013, from within the British Isles.

### 1. Mortality incident and pathology reports

Reports of wild bird mortality incidents have been solicited by the IoZ from members of the public across GB since 1993^23^ and by the RSPB since 2001. Since 2005, the RSPB and IoZ datasets have been integrated. Pathological investigations were conducted by the IoZ since 1993 and by a network of regional diagnostic laboratories from 2005–2009. Where carcasses in an adequate state of preservation were available, they were submitted for PME to the participating laboratories. In addition to a systematic visual inspection of external and internal body systems for evidence of gross abnormalities, microbiological and parasitological examinations were performed^23^,^38^.

Additional PMEs were performed by the AHVLA, 1998–2013, under the Diseases of Wildlife Scheme (DoWS) and the GB Avian Influenza in Wild Birds Surveillance (AIWBS) project, using a standard examination protocol with minor modifications to that described above.

Across laboratories, samples from a range of organs were fixed in neutral-buffered 10% formalin and processed for histopathological examination using routine methods (where permitted by the state of tissue preservation).

Real-time reverse transcription polymerase chain reaction (RRT-PCR) testing for influenza A virus, West Nile virus (WNV) and other flaviviruses was performed on pooled tissue samples from individual birds that were examined post mortem from a subset of incidents involving multiple dead birds and where the clinical history, other epidemiological information and resources, indicated this to be appropriate. Nucleic acid was extracted using commercial kits following the manufacturers’ instructions from pooled brain and kidney and screened using a pan-flavivirus RRT-PCR^39^ and a WNV-specific RRT-PCR^40^, and from pooled viscera and pooled intestinal tract from individual birds and screened using a matrix gene RRT-PCR for influenza A virus^41^,^42^.

Toxicological examinations were performed by the Wildlife Incident Investigation Scheme (WIIS^43^) on starlings submitted from a subset of incidents to exclude pesticide toxicity. Ancillary diagnostic tests were performed as appropriate, according to the incident history and relevant epidemiology: a full suite of laboratory investigations was not performed for each incident.

Incidents of starling mortality associated with water bodies were collated. Details of the site history, pathological investigation results and ancillary diagnostic test results (where available) were summarised for each incident. For the purposes of this study, a drowning incident was defined as one where one or more starling carcasses were recovered from water and where the history, PME findings and diagnostic tests performed were consistent with drowning and provided no alternative likely cause of death.

Juvenile starlings were identified on the basis of their distinct dull grey/brown plumage which is replaced in their post-juvenile moult by the characteristic dark speckled plumage of the adult. This moult typically commences in early July for first brood birds or later for second brood birds and takes around three months for completion^44^,^45^.

Details of blackbird mortality incidents where carcasses were recovered in water bodies were similarly collated.

### 2. Wild bird ring recovery data

The British and Irish Ringing Scheme, which began in 1909, is organised by the British Trust for Ornithology (BTO) with licensed ringers marking approximately 800,000 wild birds of a wide species range each year^37^. The date, location and age of the birds at the time of ringing are recorded according to a standardised protocol^46^. Approximately 15,000 of the ringed birds are subsequently reported each year, either dead or alive, by members of the public or other volunteers, with information including the date and place of finding, an estimate of age in some cases and a description of the circumstances of finding. BTO staff review this information and allocate a putative cause of death for each dead bird reported according to a standardised coding scheme^46^: “anthropogenic” (e.g. shot, trapped, pollution), “natural” (e.g. starvation, predation, disease), “drowning” and “unknown”. Drowning is recorded as the likely cause of death of birds found within water bodies.

Details of all suspected drowning reports from 1909 to 2013 inclusive were collated for all wild bird species combined, and separately for the starling and blackbird. Birds of both species that were ringed as nestlings or soon after hatching, and were subsequently recovered before October 31^st^ of the same year, were considered to be juvenile based on the timing of the post-juvenile moult. Details of the water body were summarised where available and categorised as either “natural” or “artificial”. No carcasses were available for PME from the birds reported within the ring recovery records.

## Results

### Mortality incident and pathology reports

From 1993 to 2013 inclusive, approximately 14,000 incidents of disease and/or mortality in wild birds were reported to, or collated by, the IoZ from across GB; of these approximately 120 (0.9%) involved one or more starlings. Over this period, PMEs were conducted on 3,080 birds at the IoZ, of which 69 were starlings from 38 incidents. In addition, from 1998–2013 inclusive, PMEs were conducted on wild birds submitted from 11,566 mortality incidents by the AHVLA, of which 68 were starlings from approximately 55 incidents.

Twelve incidents (from AHVLA and IoZ combined) were reported involving starling mortality in association with water. In all 12 incidents, drowning was diagnosed as the cause of mortality according to the aforementioned criteria. These incidents were distributed across England and Wales (Supplementary Table S1). Multiple birds were affected in each of the 12 reported incidents, with ≥10 birds found dead in each of 10 incidents. The smallest number of birds was found in incident 10 (Supplementary Table S1), in which two birds were found 16 days apart.

Two incidents of apparent drowning involving blackbirds were reported in 2005 and 2007. Both of these incidents occurred in autumn and involved individual blackbirds (beyond their post juvenile moult) found dead in bird baths.

Seven incidents involved only juvenile starlings one incident involved only adult starlings and a combination of juveniles and adults was involved in one incident. The age of birds involved was undetermined for three incidents. All of the starling drowning incidents occurred in May or June.

**Figure 1:**
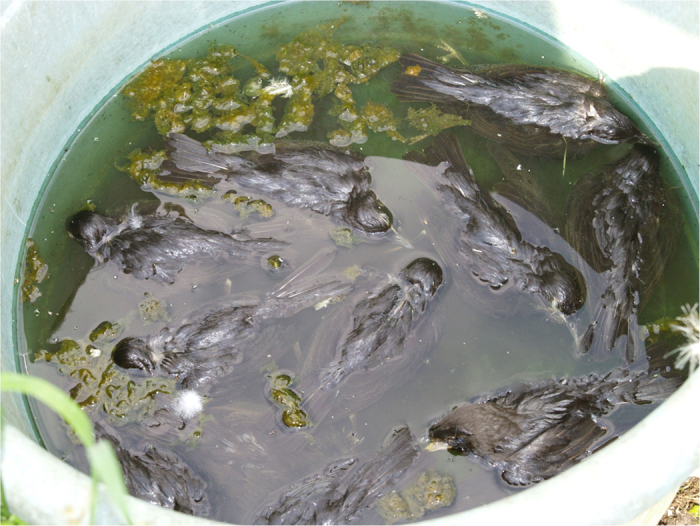
Multiple juvenile starlings (*Sturnus vulgaris*) found dead in a plastic bucket and which likely drowned.

The type of the water body varied between the incidents but the period of time that had elapsed between the incident reports and the current study precluded collection of detailed descriptive data for many cases. The water bodies involved in the starling drowning incidents are described in Table S1; they comprised garden ponds (n = 8), swimming pools (n = 2), a well (n = 1) and a garden bucket (n = 1) (Fig. 1). Four are known to have had steep sides, three had sloping sides and information about the sides of the water body was not available for five incidents. The water level was noted to be below that of the top of the water ‘container’ in three incidents and this was also presumed to be likely for the two incidents that occurred in swimming pools. Information on whether there were floating objects, or other materials (including vegetation) breaking the surface of the water, was not available in the majority of cases. Most of the incidents involved still water. Fountains were recorded at three sites and a waterfall at a fourth site, and these would have disturbed the water surface to an unknown extent when in operation. Where the time of day when carcasses were first seen was known (6 incidents), this was always in the morning. Two sites had recurrent starling mortality within a relatively short period (≤16 days) and at a further site >80 dead starlings were found over a period of several weeks. At this site, incidents of starling mortality consistent with drowning were reported in three consecutive years (2010–2012).

None of the incidents had a reported history of recent agrochemical use in the immediate vicinity of the water body, or of chemical water treatment. Two sites noted use of a weed killer (chemical identity unavailable) approximately three days before the mortality event although this was not directly in the vicinity of the water body. The water in one of the swimming pools was reported to be murky since it had not been cleaned recently. None of the sites reported visible evidence indicative of toxic algal blooms. There was no reported history of contemporaneous disease or mortality affecting sympatric domestic or wildlife species. There was no reported history of increased predator visits around the time of the incidents. Whilst some of the water bodies involved had overhanging vegetation, none were reported to have roosts of starlings above them, making it unlikely that birds had died in the trees and then fallen into the water after death.

**Figure 2:**
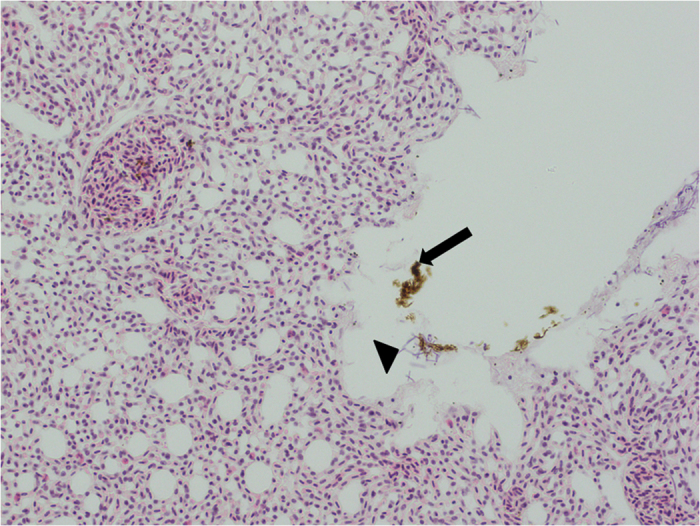
Starling (*Sturnus vulgaris*) lung Haematoxylin & Eosin (X40 objective) Respiratory atrium with focal aggregates of brown, variably staining, amorphous material (arrow) consistent with inhaled organic material. Pleomorphic bacterial rods with a morphology consistent with a *Clostridium* species are present (arrowhead), and are likely post mortem invaders.

More than 50 starlings were submitted for PME from nine apparent drowning incidents which represent at least 36.5% of all PMEs conducted on this species at IoZ and AHVLA over the study period: the results of the pathological investigations are presented in Table S2. The plumage was wet in birds from all nine incidents and aquatic invertebrates and plant matter were noted in the plumage of several birds. Birds were noted to be in good body condition in all (n = 6) incidents where this was described. Where information was available, multiple carcasses submitted from each particular incident were in a similar state of preservation indicative of a common time of death. No evidence of trauma or significant gross abnormalities was found. Only incidental bacterial and parasitic infections were detected, including *Campylobacter* sp., *Syngamus trachea* infection, and intestinal cestodes and nematodes. The state of carcass preservation precluded histopathological examination from most incidents. Histopathology was performed on tissues from seven birds from three incidents; the findings are summarised in Table S2. In one incident, brown amorphous particulate material was present within the upper airways and lung parabronchi of two of three examined birds (Fig. 2). This is consistent with organic material inhaled during drowning. No significant abnormalities were detected on microscopical examination of formalin-fixed tissues of birds from the other two incidents. RRT-PCR for WNV and other flaviviruses was performed on tissue samples collected from birds from four incidents, and for influenza A virus on tissues from birds from five incidents. All samples tested negative. Tissues from birds from four incidents were tested for exposure to agrochemical and household pesticides. No residues were detected.

Neither of the blackbirds from apparent drowning incidents was available for PME.

### Wild bird ring recovery data

Drowning was listed as the probable cause of death of 2,901 (0.35%) of the 828,924 wild birds of 79 species for which wild bird ring recoveries had been reported since 1909. Starling (n = 467) and blackbird (n = 464) were the species for which drowning was most frequently reported within the ring recovery dataset and a summary of these data is shown in Table 1. Starlings were classified as having drowned in a significantly greater proportion of ring recoveries than blackbirds, both when the entire dataset was considered (starling 1.22%; blackbird 0.87%; χ^2^_1_ = 27.5, p < 0.001) and when only those birds thought to have died as a result of natural consequences (i.e. excluding shot, trapped, polluted and records of undetermined cause) were reviewed (starling 5.04%; blackbird 3.73%; χ^2^_1_ = 22.12, p < 0.001). Juvenile birds were reported to drown more frequently than adults among both blackbirds (2.37% vs 0.72%, χ^2^_1_ = 136, P < 0.001) and starlings (3.82% vs 1.06%, χ^2^_1_ = 125, P < 0.001). Juvenile starlings were reported to drown more frequently than juvenile blackbirds, although this difference was not significant (χ^2^_1_ = 0.67, P > 0.1).

**Figure 3:**
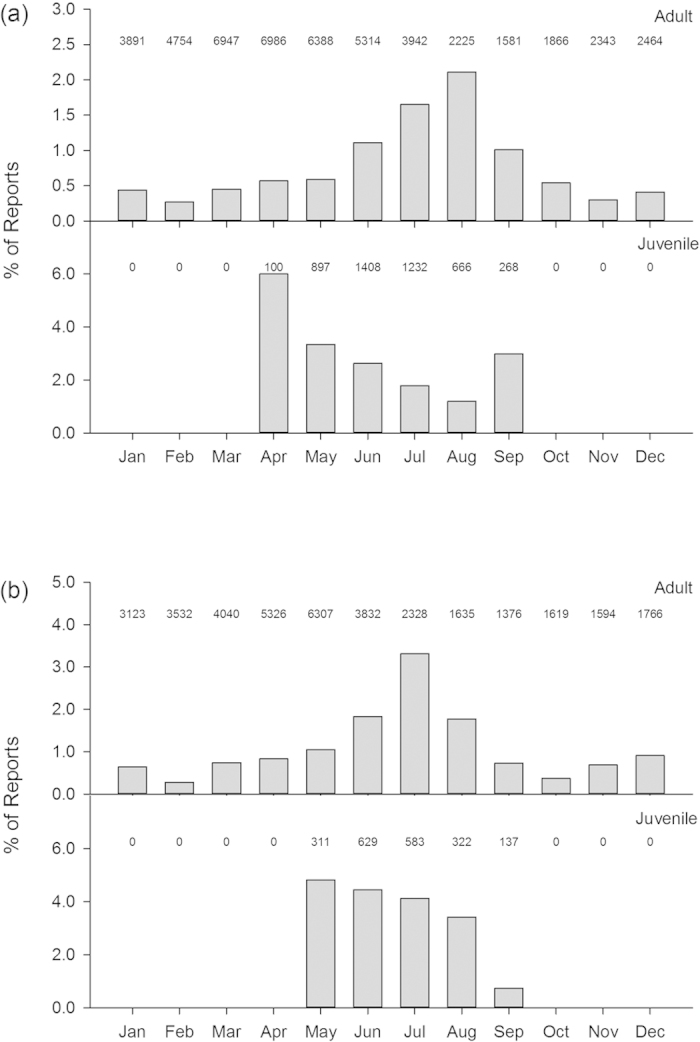
Seasonality of ring recoveries 1909–2013 for which the bird was classified as having drowned, for starling (*Sturnus vulgaris*) (**a**) and blackbird (*Turdus merula*) (**b**). The sample size for each month and age class is denoted above the plot.

**Figure 4:**
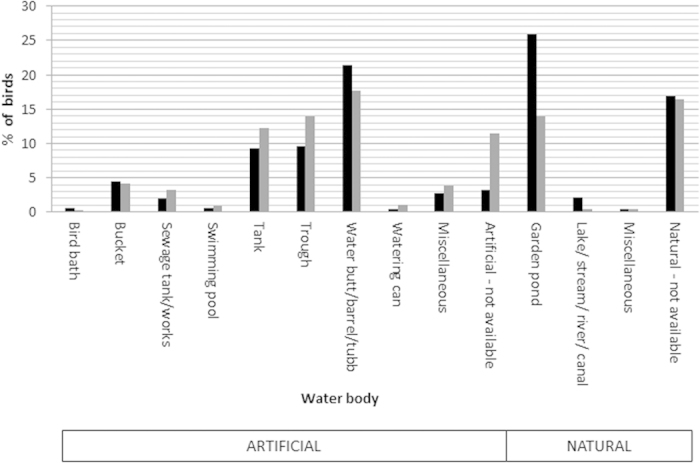
Percentage of starlings (grey) (*Sturnus vulgaris*) and blackbirds (black) (*Turdus merula*) that were recovered in water body types.

Data on the number of birds found dead per incident was not available within the ring recovery dataset. Although the relative number of reports of drowned birds remained high through the summer for juveniles, the frequency of reports peaked in the late summer for adults (Fig. 3). The water body types in which ringed starlings were found dead (Fig. 4) comprised those that were classified by the BTO as “artificial” (including bird bath, bucket, sewage tank/works, swimming pool, tank, trough, water butt/barrel/tub, watering can) and “natural” (including (garden) pond, lake/stream/river/canal) with starlings reported more frequently than blackbirds as drowning in an artificial source (χ^2^_1_ = 20.5, P < 0.001). This difference was particularly marked amongst juveniles (starling: 70% of reports coded as “artificial”, blackbird 43%, χ^2^_1_ = 12.9, P < 0.001), and less so amongst adults (starling: 69%, blackbird 58%, χ^2^_1_ = 9.22, P < 0.002).

## Discussion

### Mortality incident and pathology reports

We identified 12 mortality events involving multiple starlings consistent with drowning in Britain over the period 1993–2013 inclusive, and carcasses were examined post mortem from nine of these sites. We are not aware of any other passerine species for which mass drownings have been reported. Whilst a full suite of tests was not performed on birds from each incident, our investigations revealed no evidence for a toxic, infectious or traumatic (other than drowning) cause of death. In one incident, there was evidence of amorphous material in the respiratory tract of two birds, consistent with the inhalation of organic material during drowning.

As with all other species apart from starlings, both reports of blackbird drowning were of individual birds which may be explained by the fact that blackbirds are less gregarious than starlings and generally only associate loosely with conspecifics when foraging^47^. Starling drowning incidents almost always involved multiple birds. This might lead to reporting bias: multiple dead birds are both more likely to be seen and more likely to be reported than individual drownings. The one starling drowning event that involved two birds drowning individually over a 16 day period was observed and reported by one of the authors (BL).

The large number of starlings affected in reported drowning incidents is striking. That a single species, the starling, was involved in each of the reported multiple drowning incidents supports a species-specific behavioural cause. Apart from one incident (incident 12, Table S1) which occurred during a heat wave, there was no indication that adverse weather was implicated in the reported incidents. Whilst adult starlings were reported to be involved in a small number of reported incidents, the majority comprised only juveniles.

Starling drowning incidents occurred in May and June, following the fledging of first broods and their progression to parental independence^44^. Juvenile starlings are particularly gregarious and are frequently observed feeding and water-bathing in flocks: birds involved in these incidents may have entered the water body to drink or bathe. Following emergence from the nest, juvenile starlings have a period of high risk, whilst they learn to become independent from their parents. This is the period when they are more likely to die from predation or become victims of accidents such as “*falling into ponds, hitting windows, or road traffic accidents*”^44^. As a flocking species, juvenile and adult starlings water-bathe and drink communally, but this gregarious behaviour is infrequently observed in juvenile or adult blackbirds (R. Robinson, *pers. obs.*).

Starling drowning incidents might arise from an individual leader bird, or an entire flock, mistaking a water body as a solid surface, in a similar way that waterfowl which crash land on wet road surfaces are thought to mistake them as rivers or streams^48^. Most of the starling drowning incidents involved still water which, depending on the light and aspect, might have resulted in a reflective appearance suggestive of a solid surface. Fountains were present in at least three of the sites and a waterfall feature at one site, and these are likely to have disturbed the water surface, interrupting any reflection, but their operational status at the time of the incidents is unknown. For the incidents known to have occurred shortly after dawn, the dull light of the early morning may have altered the appearance of the water body surface.

In one incident (incident 12, Table S1), starlings continued to attempt to enter the water body once the majority of its surface had been deliberately covered over. At least three birds drowned once the water body had been partially covered. Starlings have strong flocking tendencies and overcrowding could trigger a panic response in the confined space of a small water body where jostling between birds could prevent their escape. Whilst the description of each water body was variable in quality and was often incomplete, they had steep-sided edges at several of the sites. Consequently, birds may have found it difficult to exit these water bodies. Raptors have been observed to drown when young, inexperienced birds appear to have misjudged the water depth, their plumage has become waterlogged, and they have been unable to exit steep-sided water bodies such as livestock troughs, garden tanks and water butts^35^.

In addition to inexperience, the plumage of juvenile starlings may be less water-resistant and more prone to waterlogging than birds in adult plumage, but this requires further investigation.

There are marked similarities between the described drowning events in British starlings and those observed affecting South African vultures (*Gyps africanus* and *G. coprotheres*)^49^. *Gyps* spp. vultures are also gregarious species that bathe and drink communally and their drowning incidents have likewise involved multiple birds with juveniles over-represented. These drowning incidents frequently occurred in small circular water tanks with vertical walls used in farmland habitats for domestic livestock. The majority of reservoirs in which vultures drowned were reported to be ≤75% full, therefore a physical explanation has also been put forward where birds, once immersed in the water, may have had difficulty in escaping the water body.

Climate has been proposed as a predisposing factor for vulture drowning incidents since they occur most frequently in arid regions where lack of water availability might promote reliance on anthropogenic sources (e.g. livestock troughs)^49^. In incident 12 from the current study, a recent heatwave was reported and the starlings were noted to continue to try and access the water source over a period of several days, therefore thirst may have been a factor. Whilst details of the weather were not available for the majority of the probable drowning incidents in British starlings, thirst seems an unlikely common primary driver given the typically wet British spring climate. Further study could include collation of meteorological data for historical starling drowning incidents from local weather stations. Details of the ambient conditions should be recorded in future incidents to help explore this possible predisposing factor.

It has also been hypothesised that vulture drowning incidents may involve birds that have been poisoned (e.g. with strychnine) and consequently developed excessive thirst. Further toxicological analyses are required to elucidate this theory, but there is minimal supportive evidence for this to date^49^. Resources did not permit toxicological examination of birds from many of the British starling drowning incidents and it is recommended that this be undertaken in future incidents; nevertheless there was no suspicion of intoxication on the basis of the available history or investigation findings.

### Wild bird ring recovery data

A review of the BTO ring recovery dataset showed that probable drowning incidents occur across a range of wild bird species, but most commonly involve blackbirds and starlings; drowned starlings were, however, reported significantly more frequently than drowned blackbirds. As with the starling incident reports, juvenile birds of both species were reported to drown more frequently than adults. We also found that starlings were more likely to drown in “artificial” water bodies than blackbirds, so it is possible that our results are affected by detection bias, since “artificial” water bodies are more likely to be observed by the public. However, in the BTO dataset, garden ponds were classified as “natural” water bodies which argues against such a detection bias.

As the blackbird is more commonly seen in gardens than the starling^50^, the converse bias (i.e. over-reporting of blackbird drowning incidents) might be expected. The preferential habitat use of the blackbird and starling, more frequently utilising garden and agricultural land respectively, may explain the types of water body involved in the drowning incidents with blackbird drownings more likely to occur in garden ponds and water butts/barrels/tubs and starling drownings more likely to occur in livestock troughs (see Fig. 4). From the available ringing data, however, it was not possible to determine whether the birds drowned singly or whether multiple birds were involved.

### Conclusions

Whilst the reported starling drowning incidents are noteworthy, and can cause considerable public concern when large numbers of birds are involved, our results indicate that these events are relatively infrequent and are therefore more of an animal welfare concern than conservation issue. Drowning should be included as a differential diagnosis for starling multiple mortality events, particularly for those that occur in the spring and involve juvenile birds found in water. Detailed information should be collected from future suspected starling drowning events including the distribution of carcasses found at the site, the type of water body involved, for example its dimension and construction, the presence or absence of man-made features (e.g. fountains and whether they were in operation), the time of day that it occurred, weather conditions and other descriptive site risk factors that could predispose to these incidents. That recurrent starling mortality due to drowning, rather than a single drowning episode, was observed at three of the sites in this study might indicate increased risk of occurrence associated with particular site features. Such information might inform appropriate mitigation measures that can be adopted, for example the use of floating structures or ramps that could be used to assist birds to escape from water bodies, particularly during the period of high risk (May-June).

## Additional Information

**How to cite this article**: Lawson, B. *et al.* Drowning is an apparent and unexpected recurrent cause of mass mortality of Common starlings (*Sturnus vulgaris*). *Sci. Rep.* **5**, 17020; doi: 10.1038/srep17020 (2015).

## Supplementary Material

Supplementary Information

## Figures and Tables

**Table 1:** Causes of death of ringed starlings and blackbirds reported to the British and Irish Ringing Scheme between 1909 and 2013.

	Cause of death category based on ring recovery data				Total
	Drowning Number of birds (% for each age category)	Anthropogenic Number of birds (% for each age category)	Natural Number of birds (% for each age category)	Unknown Number of birds (% for each age category)	
Blackbird					
Adult	353 (0.7)	13605 (27.9)	10937 (22.5)	23806 (48.9)	**48701**
Juvenile	111 (2.4)	1191 (26.0)	1021 (22.3)	2250 (49.2)	**4573**
Undetermined	0 (0)	12 (26.7)	7 (15.6)	26 (57.8)	**45**
**Total**	**464 (0.9)**	**14808 (27.8)**	**11965 (22.4)**	**26082 (48.9)**	**53319**
Starling					
Adult	388 (1.1)	7530 (20.8)	8195 (22.6)	20085 (55.5)	**36198**
Juvenile	79 (4.0)	306 (15.4)	595 (29.9)	1010 (50.8)	**1990**
Undetermined	0 (0)	6 (15.4)	6 (15.4)	27 (69.2)	**39**
**Total**	**467 (1.2)**	**7842 (20.5)**	**8796 (20.3)**	**21122 (55.3)**	**38227**

Table Legend: BTO staff allocate a putative cause of death for each dead bird reported according to a standardised coding scheme^46^: “anthropogenic” (e.g. shot, trapped, pollution), “natural” (e.g. starvation, predation, disease), “drowning” and “unknown”. Drowning is recorded as the likely cause of death of birds found within water bodies.
