# Supplementary material for: Drowning is an apparent and unexpected recurrent cause of mass mortality of Common starlings (Sturnus vulgaris)
Source: Sci Rep. 2015 Nov 25;5:17020. doi: 10.1038/srep17020 (PMC4658497; doi:10.1038/srep17020)
Supplement: Supplementary Information [file srep17020-s1.pdf]

Title: Drowning is an apparent and unexpected recurrent cause of mass mortality of Common starlings

Author Names:

Becki Lawson*[1], J. Paul Duff[2], Katie M. Beckmann[1], Julian **Chantrey**[3]**, Kirsi M. Peck**[4]**, Richard M. Irvine**[5]**, Robert A. Robinson**[6]**,** Andrew A. Cunningham[1]

Supplementary Table 1:

Summary of Common starling incidents for which the cause of death was considered to be drowning.

**Incident Number**	**Date**	**County, Country**	**Number of starlings**	**Age of birds**	**Water body description/ source of water**	**Notes**	**Time of day of suspected occurrence**
1	May 1994	Kent, England	18 (16 on first day, 2 on second day).	Adults	Garden pond<br><br>New (present for 3 weeks only) fiberglass pond, 6 x 3 ft x 2.5ft deep. Small water lily plant in water. Raised ridge around edge of pond that birds can perch on.	Overhung by lilac tree but not large or known to be used as a roost.	Morning (between 8 and 10am).

Incident Number	Date	County, Country	Number of starlings	Age of birds	Water body description/ source of water	Notes	Time of day of suspected occurrence
2	May 1995	Bedfordshire, England	>7	Juveniles	Garden pond?	No further details available	Not available.
3	May 1995	Essex, England	33	Unknown	Swimming pool.<br><br>Circa 3.5 m diameter. Water surface circa 15 cm below the rim. Water was murky. Nothing floating within the water.	No nearby trees for roost.	Not available.
4	May 2005	West Yorkshire, England	28	Juveniles	Garden pond<br><br>Circa 6 months old.	Pond naturally colonised with frog spawn. No recent work or treatment to pond. Trees in garden but no roosts above pond.	Not available.
5	May 2006	Essex, England	41	Juveniles (recently fledged)	Garden pond.<br><br>Established pond 2m diameter and shallow. Not	Trees around pond.	Found dead in the morning.

Incident Number	Date	County, Country	Number of starlings	Age of birds	Water body description/ source of water	Notes	Time of day of suspected occurrence
					steep sided.		
6	May 2006	Essex, England	23	Juveniles	Garden pond.<br><br>Established, small plastic moulded pond 1.75 m diameter with waterfall Rocks in water and not steep sided.	Other birds use pond to drink and bathe with no problem.<br>Nature reserve behind house.	Found dead in morning.
7	May 2006	Cambridgeshire, England	11	Juveniles	Swimming pool<br><br>Established pool in school.	No further details available.	Not available.
8	May 2007	East Sussex, England	18 submitted	Unknown	Garden pond.<br><br>Small circular pond with fountain and vertical sides.	No further details available.	Not available.
9	May 2008	Wiltshire, England	12	Juveniles	Plastic bucket.<br><br>Bucket in garden containing	No known starling roost in semi-rural garden; starlings use area of neighbouring land.	Not available.

**Incident Number**	**Date**	**County, Country**	**Number of starlings**	**Age of birds**	**Water body description/ source of water**	**Notes**	**Time of day of suspected occurrence**
					circa 10 cm of water. Steep sides.	No recent chemical use reported. No overhead objects.	
10	June 2008	Greater London, England	2 (single birds on non-consecutive days at 16 day interval).	Juveniles	Garden well.<br><br>Ornamental well made of bricks with central fountain. 120cm diameter and circa 30 cm deep. Water surface c. 15 cm below rim. Steep sides.	No further details available.	One found early morning around 9am and second bird around 9pm.
11	June 2010	Glamorgan, Wales	19	Unknown	Garden pond.<br><br>Small ornamental pond with steep sides.	No further details available.	Carcasses found in rigor in the morning after return from 3 week holiday.
12	May 2011	Bedfordshire, England	>80<br><br>The incident was ongoing for approx. 2 weeks (mid May	Mainly adults, few juveniles	Garden pond.<br><br>Fountain present. Dimensions	Starlings roost in tree nearby. No sick birds seen in garden and no carcasses found	Found most often in early morning.

Incident Number	Date	County, Country	Number of starlings	Age of birds	Water body description/ source of water	Notes	Time of day of suspected occurrence
			to early June 2011). Over 40 of these birds were found dead within a single weekend.<br><br>A single bird was found alive in the water and apparently recovered when taken onto land.<br><br>A similar incident occurred the previous year (2010) with circa five dead starlings found in the same pond over a period of weeks.		circa 1.2 x 2.7 x 0.9 m m deep. Pond built around 2002. Rocks over smooth pond liner. Slightly sloping sides.<br><br>Water lilies present in the pond.	anywhere other than floating in pond.<br>Birds were observed struggling to get into the water even when the pond had been covered over (initially with chicken wire and then wooden pallets): at least three birds were found in the water after it had been covered over. No reported use of pesticides. This incident was reported during a heat wave.<br><br>Further mortality occurred in 2012 with circa 15 dead starlings found in groups of 3-4 dead	

**Incident Number**	**Date**	**County, Country**	**Number of starlings**	**Age of birds**	**Water body description/ source of water**	**Notes**	**Time of day of suspected occurrence**
						birds.<br>No mortality in 2013 or 2014.	

Supplementary Table 2:

Summary of Common starling post mortem examinations and ancillary diagnostic test results

**Incident Number**	**Number of starlings examined**	**Body condition**	**Gross examination**	**Microbiology (number examined given in brackets)**	**Histopathology (number examined given in brackets)**	**Virology (number examined given in brackets)**	**Toxicology (number examined given in brackets)**
2	3	Good<br><br>Ample fat deposits and good pectoral muscle condition.	Each carcass was wet; aquatic beetle in plumage of one bird.<br>No evidence of trauma, disease or significant macroscopic abnormality detected.	n.d.	n.d.	n.d.	n.d
4	3	Good	Each carcass was wet. No evidence of trauma, disease or significant macroscopic abnormality detected.	Liver (3): no significant isolates<br><br>Small intestinal (SI) contents (3): *Campylobacter* sp. isolated	Preservation: moderate autolysis<br><br>Small intestine (2): intralumenal cestodes<br><br>Heart, liver,	n.d.	Tissues submitted to WIIS for alphachloralose, carbamate and metaldehyde testing (3): no residues detected.

Incident Number	Number of starlings examined	Body condition	Gross examination	Microbiology (number examined given in brackets)	Histopathology (number examined given in brackets)	Virology (number examined given in brackets)	Toxicology (number examined given in brackets)
			Incidental endoparasites (*Syngamus trachea* in 2/3 cases) Leatherjacket in plumage of one bird.		lung, kidney, spleen, CNS, pancreas, adrenal (2): NLD		
5	20	Good	All submitted birds were soaked with pond weed. Food in gizzards. No obvious lesions in respiratory tract and other systems. Autolysed carcasses.	Pooled small intestinal contents screened for *Salmonella* sp. only: negative	n.d.	AIV (8): negative	Tissues submitted to WIIS for alphachloralose, carbamate and metaldehyde testing (≥2): no residues detected.
6	5	Good	All submitted with soaked feathers. Autolysed carcasses. No gross	Pooled small intestinal contents screened for *Salmonella* sp. only: negative	n.d.	AIV and WNV on pooled tissues (2): negative	Tissues submitted to WIIS for alphachloralose, carbamate and metaldehyde

Incident Number	Number of starlings examined	Body condition	Gross examination	Microbiology (number examined given in brackets)	Histopathology (number examined given in brackets)	Virology (number examined given in brackets)	Toxicology (number examined given in brackets)
			abnormalities detected in any body system.				testing (≥2): no residues detected.
7	11	Not given	Submitted sodden, autolysed, fly eggs deposited. No gross lesions detected in body systems but birds autolytic.	Pooled small intestinal contents screened for *Salmonella* sp. only: negative	n.d .	AIV and WNV on pooled tissues (≥2): negative	
8	3 (18 received)	Not given	Submitted with sodden feathers. Autolysed. No gross lesions detected, food present in gizzards.	SI contents (2): no significant isolates.	n.d.	AIV and WNV (3): negative	n.d.
10	2	Good<br><br>(subcutaneous and coronary	Each carcass was wet. No evidence of trauma or	Liver (2), small intestine (2): no significant	Preservation: moderate autolysis	n.d.	n.d.

Incident Number	Number of starlings examined	Body condition	Gross examination	Microbiology (number examined given in brackets)	Histopathology (number examined given in brackets)	Virology (number examined given in brackets)	Toxicology (number examined given in brackets)
		band fat deposits present)	significant abnormality. Lungs congested in one bird; lungs pale enlarged and buoyant in formalin for the second case. Incidental endoparasites (cestode and nematode in small intestinal contents; both birds). SI contents parasitology negative in both cases.	isolates.	Lungs: diffuse to moderate vascular congestion (2) Liver: multifocal moderate hepatocyte brown granular cytoplasmic pigment (haemosiderosis) (2) Heart and skeletal muscle (2) NLD. Kidney, brain, spleen, trachea and Bursa of Fabricius (1) NLD.		
11	19 submitted, number examined not given.	Not given	Birds soaked on submission. No	n.d.	n.d.	AIV and WNV on pooled tissues (≥2: negative	n.d.

Incident Number	Number of starlings examined	Body condition	Gross examination	Microbiology (number examined given in brackets)	Histopathology (number examined given in brackets)	Virology (number examined given in brackets)	Toxicology (number examined given in brackets)
			pathological lesions seen.				
12	3	Good<br><br>Ample subcutaneous and coronary band fat deposits.	Soaked plumage, and aquatic larvae on each bird.<br><br>Marked bilateral lung congestion in each bird.<br><br>Numerous cestode ova in SI contents of one bird. Numerous ascarid-type parasites in SI contents of second bird. Third negative for parasites.	Liver (2)<br>Lung (1)<br>SI contents (2): no significant isolates.	Preservation: mild to moderate autolysis (3)<br>Lungs: diffuse moderate vascular congestion (3).<br><br>Occasional brown particulate matter is present within airways and parabronchi accompanied by shed epithelial debris and bacterial rods, likely post mortem invaders (Figure 2) (2)<br>Liver: multifocal moderate hepatocyte brown granular	n.d.	Tissues submitted to WIIS for alphachloralose, carbamate, metaldehyde and rodenticide testing (3): no residues detected.

Incident Number	Number of starlings examined	Body condition	Gross examination	Microbiology (number examined given in brackets)	Histopathology (number examined given in brackets)	Virology (number examined given in brackets)	Toxicology (number examined given in brackets)
					cytoplasmic pigment (haemosiderosis) (3)<br>SI: Nematodes within intestinal lumen and within the muscularis and connective tissue of the intestinal wall associated with multifocal mixed inflammatory cell infiltrate. Acute myocarditis in right ventricular free wall (1).<br><br>Heart (2), skeletal muscle (3), kidney (3), Bursa of Fabricius (3), trachea (2), brain		

Incident Number	Number of starlings examined	Body condition	Gross examination	Microbiology (number examined given in brackets)	Histopathology (number examined given in brackets)	Virology (number examined given in brackets)	Toxicology (number examined given in brackets)
					(2): NLD.		

Footer:

n.d. = not done

NLD = no lesions detected
